# Determination and Verification of GISSMO Fracture Properties of Bolts Used in Radioactive Waste Transport Containers

**DOI:** 10.3390/ma15051893

**Published:** 2022-03-03

**Authors:** Bonjoon Gu, Jongmin Lim, Seokmoo Hong

**Affiliations:** 1 Department of Future Convergence Engineering, Kongju National University, Cheonan 31080, Korea; bonjoon99@smail.kongju.ac.kr; 2 Korea Atomic Energy Research Institute, Daejeon 34057, Korea; jmlim86@kaeri.re.kr; 3 Department of Future Automotive Engineering, Kongju National University, Cheonan 31080, Korea

**Keywords:** bolt, damage, digital image correlation, finite element analysis, GISSMO

## Abstract

Transport containers for radioactive materials should withstand drop tests according to the regulations. In order to prevent a loss or dispersal of the internal radioactive materials in the drop tests, a tightening of the lid of the transport container should be maintained. The opening of the lid, due to the drop impact, might cause the dispersion of internal contents or a loss of shielding performance. Thus, it is crucial to predict damage to the fastening bolt and its fracture. In this study, the damage parameters of the fastening bolt were acquired, and its fracture was predicted using the generalized incremental stress state-dependent damage model (GISSMO), a phenomenological damage model. Since the dedicated transport container is large and heavy, various jigs that can simulate the fall of the container were designed, and the accuracy of fracture prediction was verified. Digital image correlation (DIC) was introduced for the accurate measurement of the displacement, and load–displacement data for tensile, shear, and combined loads were successfully acquired. Finally, the load–displacement curve of the finite element analysis (FEA) with GISSMO until the point of the bolt fracture was compared with the curve obtained from the experiment, where a good agreement was observed.

## 1. Introduction

**Figure 1:**
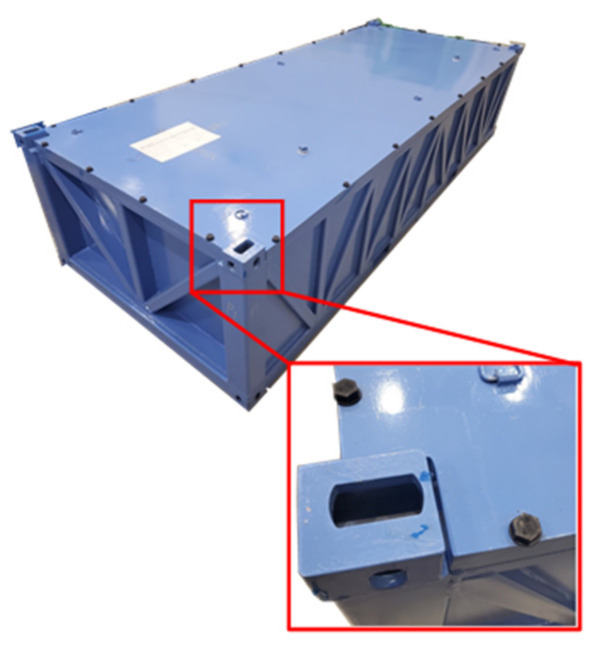
IP-type radioactive waste transport container.

In order to prevent radiation exposures during transportation, radioactive materials should be transported in a transport container that satisfies the regulations [1,2]. In general, radioactive waste transport containers are made of metals, and the upper and lower parts of them are fastened with bolts to prevent the dispersion of radioactive waste to the outside (Figure 1).

Radioactive waste is generated the most when dismantling a nuclear power plant, and is 10 to 200 times larger than that generated during power plant operation [3]. Marsh et al. [4] investigated canister characteristics for the engineering barriers of geological disposal facilities to dispose of highly radioactive waste, to research the importance of the role and performance of the barrier lifespan. Pérot et al. [5] performed the characterization and quantification of radioactive waste using non-destructive measurement techniques, high-energy photon imaging (radiography, tomography) and digital auto-radiography. The process of safely transporting the generated radioactive waste is very important. According to the regulations [1,2], the transport container should be subjected to a drop test that simulates various accidents that might occur during the transportation. In order to prevent loss or dispersal of the internal radioactive materials in the drop tests, a tightening of the lid of the transport container should be maintained. An elastomer-based seal is generally installed between the bolt-tight lid and the container to prevent leakage of the contents. If the seal opens more than the limit due to deformation of the lid or fracture of the bolt due to the drop impact, the internal contents may be dispersed or the shielding performance may deteriorate. The opening of the lid due to the drop impact might cause the dispersion of internal contents or loss of shielding performance.

Therefore, it is important to predict the fracture of the bolts fastened to radioactive waste transport containers. For bolts used in the transport container of radioactive materials, the impact load that can occur during transport is applied at a very high level compared to the commercial bolt design load. Applying bolt design according to that level of design code is too conservative and can lead to overdesign. Therefore, it is necessary to develop a fracture model capable of a realistic evaluation of bolt fracture. As for studies related to bolt fracture, Cai et al. [6] predicted the fracture behavior of bolted joints at a high temperature by using the Bao–Wierzbicki model by correcting the damage parameters. Liao et al. [7] evaluated the applicability of the Gurson–Tvergaard–Needleman model and the progressive damage model by investigating damage progression and pore generation to predict the fracture of threaded bolts. Many models have been developed to predict damage where the damage occurs when the maximum tensile principal stress reaches the threshold value, according to the Cockcroft–Latham model [8]. The Brozzo model, a modified model of the Cockcroft–Latham model, expresses damage in terms of principal stress and hydrostatic stress [9]. Additionally, there exists the Oyane model [10], a model based on the volumetric deformation limit; the Johnson–Cook model, which is based on the hardening law and Mises plasticity [11]; and the Gurson model [12], a model based on porous plasticity.

Many studies have been conducted on radioactive waste treatment facilities and containers, but there have been no studies for predicting the fracture of bolts used in fastening transport containers. In addition, it is difficult to predict the fracture of bolts only with a general uniaxial tensile test. Necessarily, according to triaxiality, fracture prediction should be made by considering both tensile and shear stress states. Therefore, in this study, the fracture of the bolts used for radioactive waste transport containers was predicted using the generalized incremental stress state-dependent damage model (GISSMO), one of the damage models. Uniaxial, shear, and plane strain specimens were prepared to conduct the tensile test, and necking, fracture strain and GISSMO damage parameters were obtained according to the stress state using LS-DYNA and LS-OPT to acquire the GISSMO parameters. Next, jigs that can reach the stress state at the moment when the bolt fractures due to the drop of the transport container were designed to simulate the situation, and this was used to verify the accuracy of the fracture prediction. At this time, the digital image correlation (DIC) measurement method was introduced to measure the accurate displacement of the jig. The measurements were compared with the load–displacement curve obtained through finite element analysis (FEA) until the bolt fractured to verify the reliability of the GISSMO parameters.

## 2. Acquisition of Bolt Properties through Tensile Tests

### 2.1. Specimen Design for Different Stress States

The tensile test was conducted to acquire the bolt properties to be analyzed. SNB7, alloy steel commonly used because of its high-temperature properties, was used as a material, and its basic properties are shown in Table 1.

**Figure 2:**
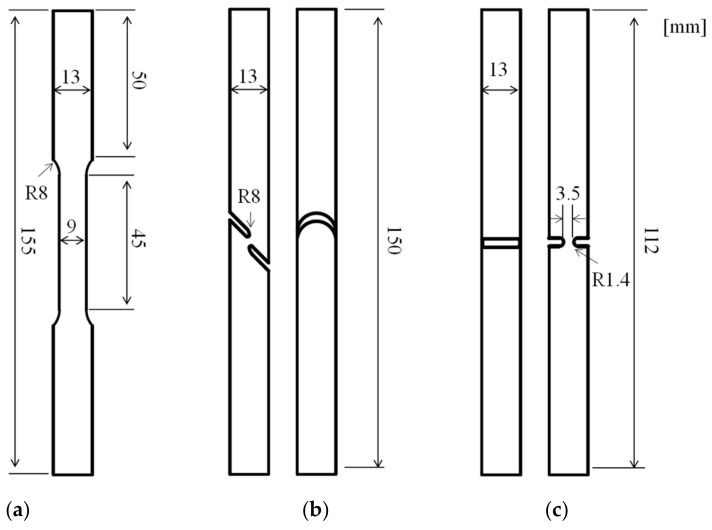
Designed specimens (**a**) uniaxial tension (UT), (**b**) shear (SH45), (**c**) plane strain (N5).

To consider the various stress states acting on the bolt when the transport container falls, three specimens in the uniaxial tension (UT), shear (SH45), and plain strain (N5) states, except for the compressive stress state, were designed using the round bar shape. The accurate dimensions and geometry of the specimens are shown in Figure 2. To determine whether the designed specimens reached an appropriate stress state, the triaxiality (*η*) was examined from the initial state to the point of fracture. *η* is defined as the value obtained by dividing the mean stress by the equivalent stress, as shown in Equation (1) [13].

The stress state is expressed using the value obtained by dividing the mean stress by the equivalent stress using Equation (1), together with the von Mises stress equation. Where *σ*_1_, *σ*_2_, and *σ*_3_ are the principal stresses in each direction, and representative *η* according to the stress state is shown in Table 2.

(1) $$\eta =\frac{\sigma_{m}}{\sigma_{v}}=\frac{\frac{1}{3}\left(\sigma_{1}+\sigma_{2}+\sigma_{3}\right)}{\sqrt{\frac{{\left(\sigma_{1}-\sigma_{2}\right)}^{2}+{\left(\sigma_{2}-\sigma_{3}\right)}^{2}+{\left(\sigma_{3}-\sigma_{1}\right)}^{2}}{2}}}$$

**Figure 3:**
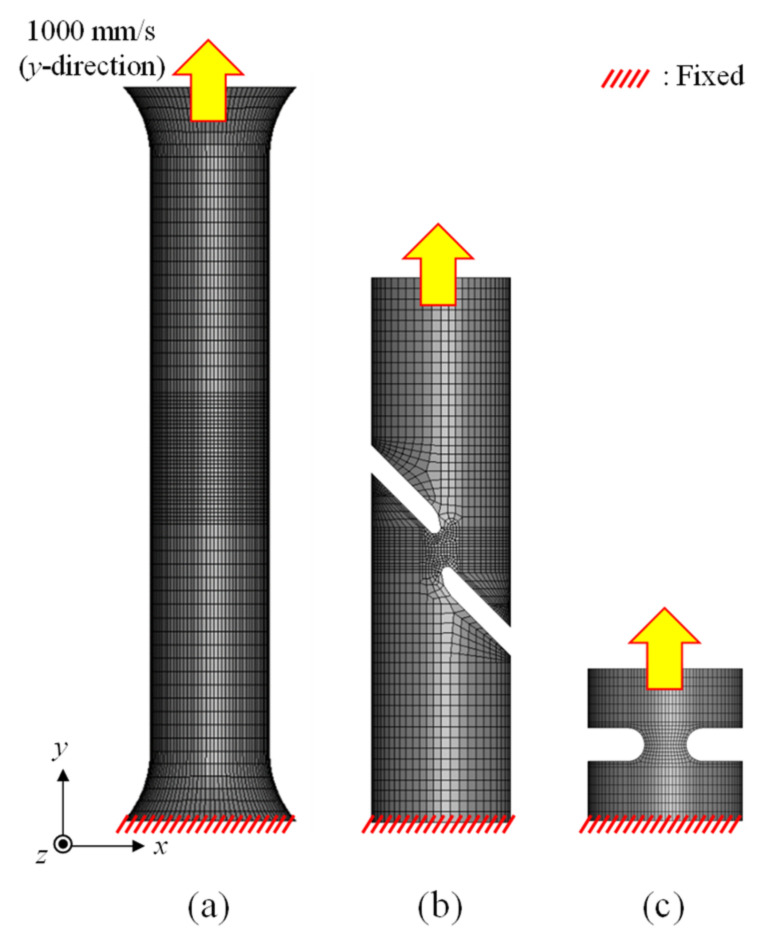
FE modeling of specimens (**a**) UT (**b**) SH45 (**c**) N5.

**Figure 4:**
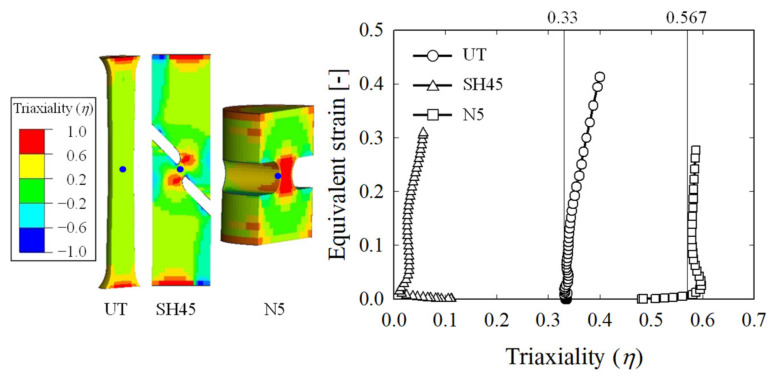
Triaxiality *η* depending on the type of specimen.

The modeling of each specimen is shown in Figure 3, and all 8-node hexahedral elements were used for the element types used in the FE model. The left-hand figure in Figure 4 shows the cross-section of each specimen in the FEA results, while the right-hand figure shows the triaxiality of each specimen according to the strain. All FEA results used in this paper are calculated by LS-DYNA using NVIDIA DGX Station (Future Automotive Intelligent Electronics Core Technology Center, Cheonan, Korea). In the case of UT, *η* initially remained at 1/3 but increased to approximately 0.4 at the fracture point due to local deformation after necking. For the N5 specimen, however, *η* remained approximately constant at 0.567 despite plastic deformation. In the case of the SH45 specimen, *η* gradually increased from the initial state to the fracture, and similar results were also shown in previous studies [14,15].

### 2.2. Acquisition of Material Properties Using DIC

**Figure 5:**
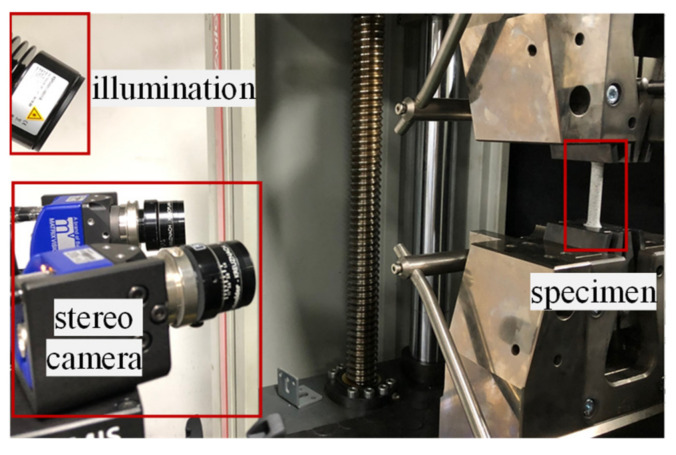
Acquisition of local deformations and load using DIC.

When conducting the tensile test, it is possible to measure local deformations until the point of the fracture without a separate strain gauge using DIC. Figure 5 shows the DIC system. Zwick Roell’s Z100 (Zwick Roell Group, Ulm, Germany) was used for the load measurement, and GOM ARAMIS (GOM, GmbH, Brunswick, Germany) [16] was used as the DIC equipment. Table 3 and Table 4 show the detailed specifications of the equipment used.

The test was conducted three times per specimen to examine repeatability, and the load–displacement curve obtained from the UT specimen was converted into a stress-strain curve. Since the change in the cross-sectional area can be calculated from the change in width or length, the nominal stress and nominal strain are defined using Equations (2) and (3), respectively, such that:

(2) $$\sigma_{e}={F/A}_{0}$$

(3) $$\varepsilon_{e}=\Delta L/L_{0}$$

where *A*_0_ is the initial cross-sectional area, ∆*L* is the changed gauge length, and *L*_0_ is the initial gauge length. When a uniform elongation is assumed for the nominal stress and nominal strain, and conservation of the volume during plastic deformation is considered, the true stress and true strain can be calculated using Equations (4) and (5), respectively.

(4) $$\sigma_{t}=\sigma_{e}\left(1+\varepsilon_{e}\right)$$

(5) $$\varepsilon_{t}=\ln\left(1+\varepsilon_{e}\right)$$

**Figure 6:**
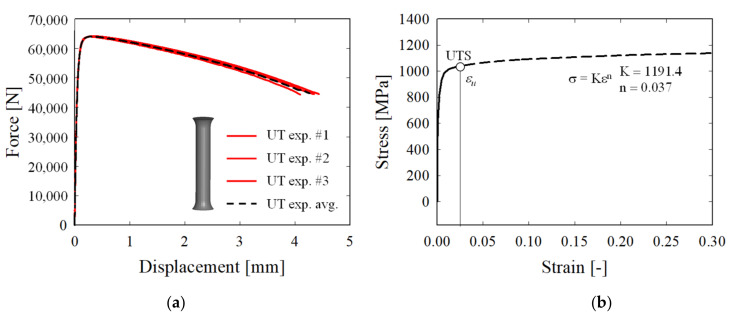
(**a**) Force–displacement curve of the UT specimen. (**b**) True stress-strain curve up to the maximum force, denoted UTS, followed by extrapolation using the Hollomon equation.

**Figure 7:**
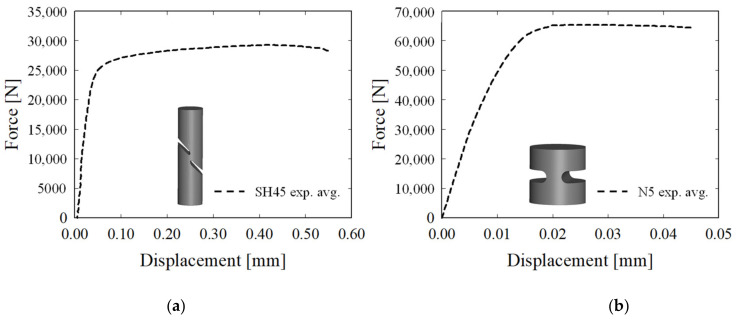
(**a**) Force–displacement curve of the SH45 specimen. (**b**) Force–displacement curve of the N5 specimen.

Figure 6a shows the load–displacement curve of the UT specimen. Since the flow stress obtained using Equations (4) and (5) is valid only when the curve is transformed up to the ultimate tensile strength (UTS), the curve beyond the UTS was extrapolated using the Hollomon equation, as shown in Figure 6b [17]. The tensile test was conducted in the same way as with the UT specimen. Figure 7a shows the average load–displacement curve of SH45, while Figure 7b shows that of N5.

## 3. Acquisition of GISSMO Damage Parameters

### 3.1. GISSMO

GISSMO, a phenomenological damage model, can have a very practical approach to damage prediction [18,19]. The forming intensity *F* and the damage parameter *D* are expressed as a weight function dependent on *ε_eq_*, an equivalent strain that initiates necking; *ε_f_*, a strain in the event of fracture; and triaxiality *η*.

(6) $$F_{i+1}=\int_{0}^{\varepsilon_{eq}}\frac{m}{\varepsilon_{u}\left(\eta i\right)}F_{i}{}^{\left(1-\frac{1}{m}\right)}d\varepsilon_{eq}$$

(7) $$D_{i+1}=\int_{0}^{\varepsilon_{eq}}\frac{m}{\varepsilon_{f}\left(\eta i\right)}D_{i}{}^{\left(1-\frac{1}{m}\right)}d\varepsilon_{eq}$$

**Figure 8:**
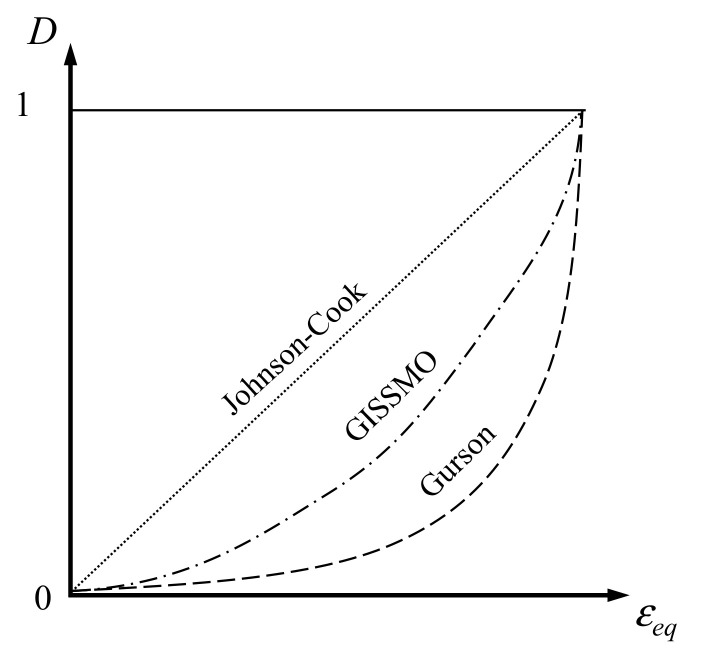
Comparison of damage progression in three damage models.

The initial *F* value was set to 10^−20^, and expressed as *i* and *i* + 1 to represent the previous and present stages of the FEA. The parameter *m*, a damage evolution index, is equal to or higher than 1. When *m* = 1 holds, Equation (6) follows the model proposed by Johnson–Cook [11]. As *m* increases, it is closer to the damage evolution model of Gurson [12], as shown in Figure 8. For GISSMO, damage progression is determined by damage parameters.

(8) $$\tilde{\sigma }=\sigma \left[1-{\left(\frac{D-D_{c}}{1-D_{c}}\right)}^{f}\right]$$

When the present *F* value reaches 1, at which diffusion necking begins, *D* is calculated using Equation (7). From this point onwards, the damage is the relational expression of Equation (8), and the stress tensor decreases. Here, *D_c_* is defined as the *D* value at which instability begins as *F* reaches 1. The fading index *f* was introduced to solve the mesh dependence problem in terms of damage progression after the critical region. For *f* = 1 and *D_c_* = 0, the Lemaitre definition of the effective stress tensor was reached. When *D* = 1 holds, it represents damage. As can be seen from Equation (7), *D* begins to be a value other than zero after yielding, but *D* > 0 does not mean that the performance of the material has deteriorated. To solve the mesh dependence problem, dependency on a specific element length was created considering the amount of energy dissipated during the fadeout of the element [20].

### 3.2. Determination of Damage Parameters

**Figure 9:**
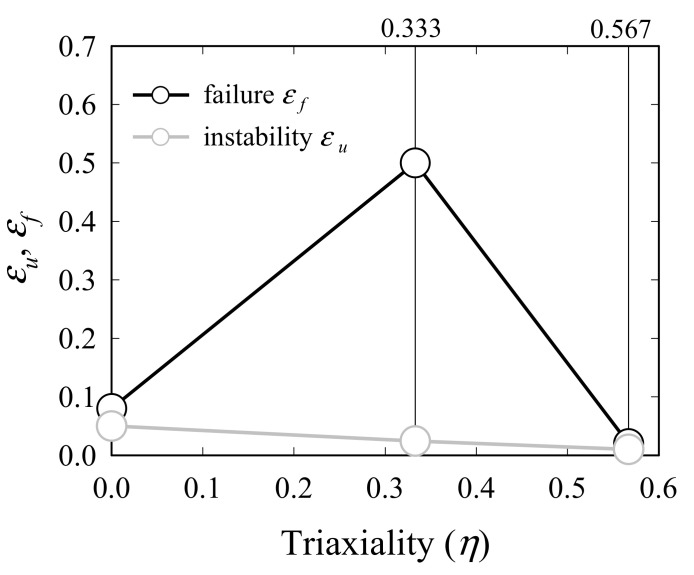
Triaxiality failure diagram (TFD).

GISSMO parameters are determined using LS-OPT, an optimal design software program [21]. After obtaining a load–displacement curve that represents the uniaxial, shear, and plane strain stress states of the UT, SH45 and N5 specimens, the GISSMO parameters are varied within a specified range and determined by minimizing the difference between the load–displacement curve calculated through analysis and that obtained from the experiment. A mathematical prediction model is constructed from the initial value in the specified range using the radial basis function networks (RBFN) algorithm to conduct the analysis and the adaptive simulated annealing (ASA) algorithm is used for curve mapping [21]. In the case of the fracture strain, the local strain measured from DIC is regarded as the initial fracture strain, and its convergence is determined by considering the specified error after comparing the load–displacement curve extracted from analysis with that of the experiment, together with other parameters. Table 5 and Figure 9 show the GISSMO parameters and triaxiality failure diagram (TFD) determined through optimization, where *ε_u_* is the necking strain and *ε_f_* is the fracture strain.

**Figure 10:**
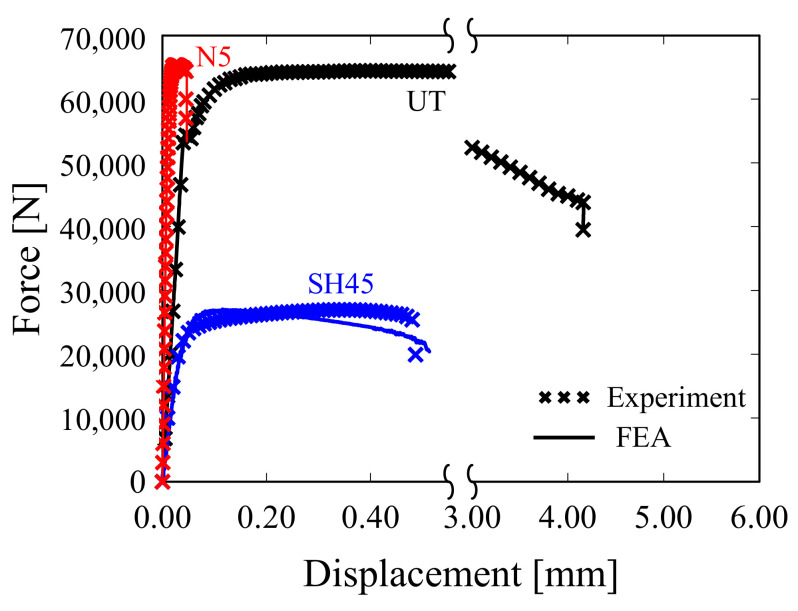
FEA and experimental force–displacement curve achieved when applying the obtained GISSMO parameters.

**Figure 11:**
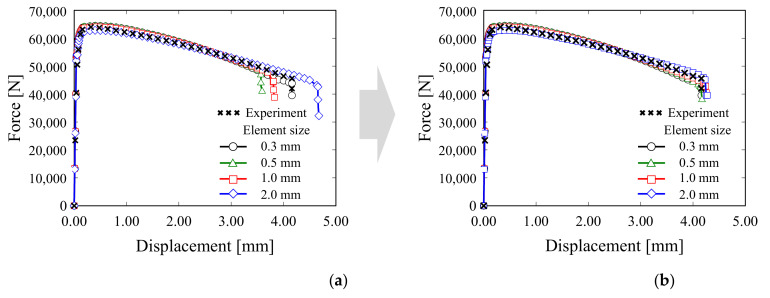
(**a**) Mesh-dependent results before mesh regularization. (**b**) Mesh-independent results using mesh regularization.

Figure 10 shows the load–displacement curves of the UT, SH45 and N5 specimens obtained from the experiment and analysis when applying the acquired GISSMO parameters. For all three specimens, the load–displacement curve resulting from the analysis was in good agreement with the experimental curve. In addition, the acquired GISSMO parameters can be applied regardless of the element size through mesh regularization. Figure 11a shows the curve according to the element size before the application of mesh regularization. Notably, differences between the curves are evident. In Figure 11b, the same results are observed regardless of the element size, acquired through mesh regularization, thereby securing mesh size independence.

## 4. Verification of Fracture Properties of Bolts Used in Radioactive Waste Transport Containers

It is necessary to physically drop the radioactive waste transport container to verify the fracture properties of the bolts used within the container. However, it is difficult to conduct this drop test due to time and costs because the container is large and weighs tens of tons. Therefore, in this study, the stress state in the event of the fracture of the actual bolt was divided into the shear stress state and combined stress state, and a jig was prepared for each state to simulate the situation.

### 4.1. Finite Element Analysis

#### 4.1.1. Boundary Conditions

**Figure 12:**
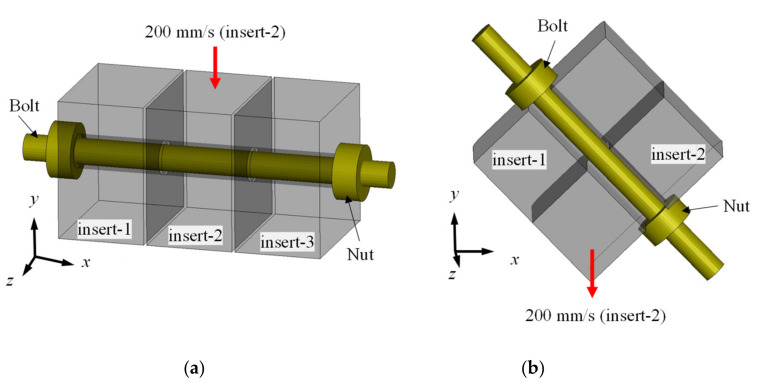
FE modeling (**a**) 90° shear jig (**b**) 45° combined jig.

The jigs for verification were designed to simulate the stress state that can be achieved in the event of the bolt fracture. Two jigs were designed, and FEA was conducted by performing FE modeling for the vertical shear jig and the 45° combined jig, as shown in Figure 12a,b, respectively. Inserts 1 to 3 were assumed as rigid bodies to examine only the deformation of the bolt, without considering the deformation of the jig, and only the working part was simplified. Since the vertical shear jig is a model of the induction of shear stress, the displacement and rotation of inserts 1 and 3 in the *x*, *y* and *z* directions were fixed, and a load condition in the −*y*-direction was applied to insert 2. For the 45° combined jig that induces tensile and plane strain, insert 1 was fixed, and the same load as that of the vertical shear jig was applied to insert 2. In the case of the M10 bolt fastened to each jig for verification, the thread was simplified to a root diameter of 8.5 mm, considering the thread pitch in the analysis model to shorten the analysis time. A load–displacement curve was obtained by extracting the *y*-direction force acting on insert 2 and its displacement.

#### 4.1.2. FEA Results

**Figure 13:**
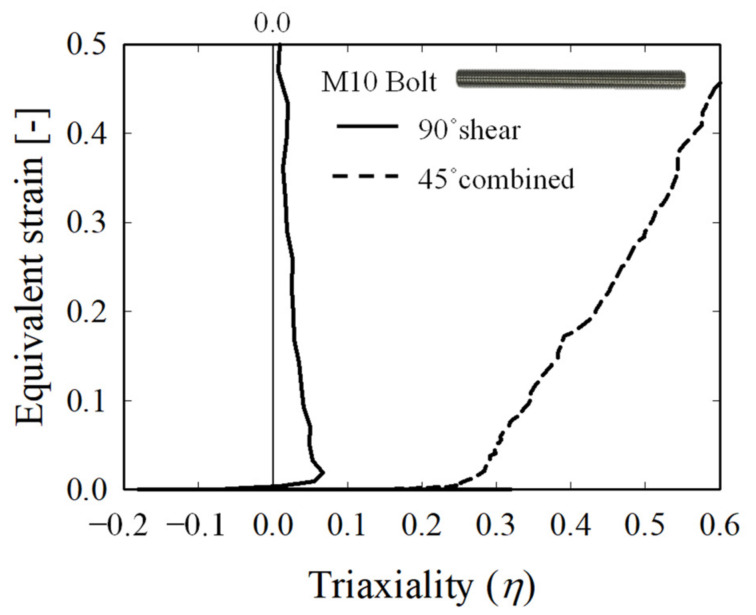
Change of bolt shear surface *η* in the designed jig.

Under the aforementioned conditions, *η* was extracted from the central element of the bolt fracture section through the FEA of the 90° shear and 45° combined jig tests, and the results are shown in Figure 13. As shown in the figure, in the case of the 90° shear jig test, a value close to *η* = 0 remained until the point of fracture. For the 45° combined jig, *η* gradually increased from 0.33, a uniaxial stress state, to the 0.4–0.6 range, a plane strain stress state. This confirms the combined load state and shows that it is possible to induce shear, tensile, and plane strain stress states through *η* secured from two jigs.

### 4.2. Bolt Fracture Test

**Figure 14:**
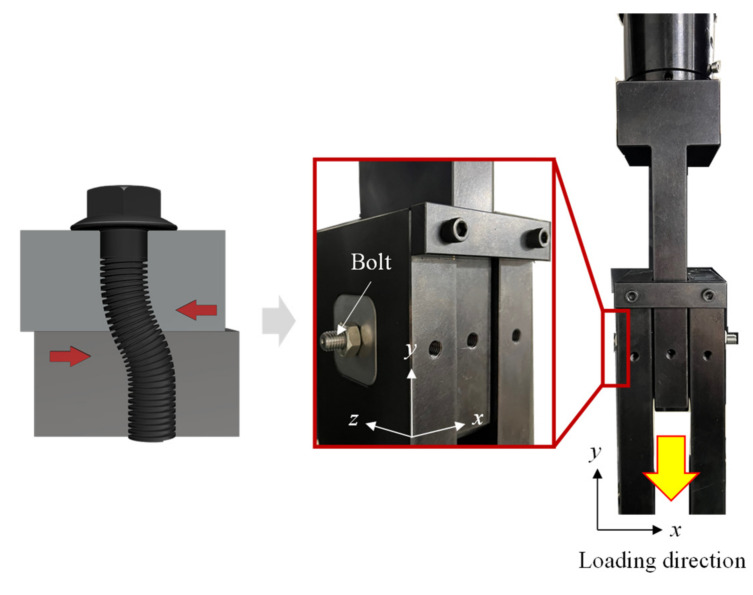
The 90° shear jig designed for vertical shear fracture.

**Figure 15:**
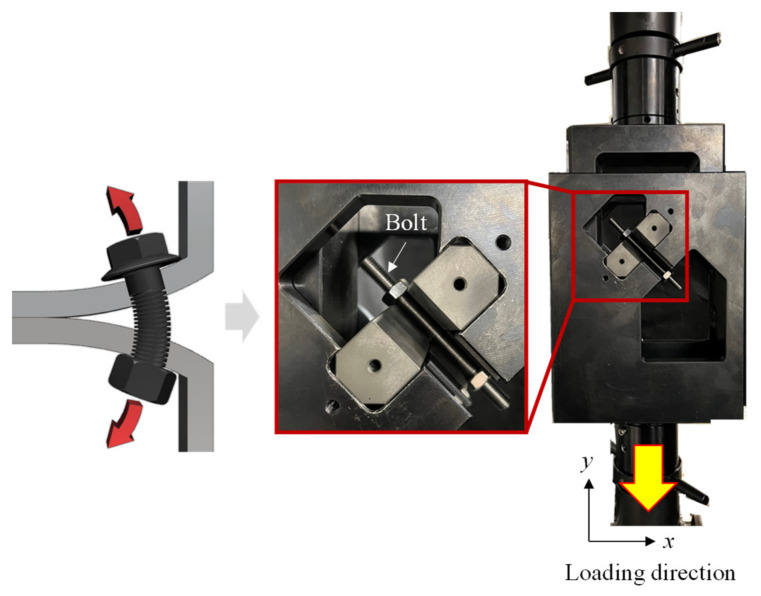
The 45° combined jig designed for combined load fracture.

Figure 14 and Figure 15 show the bolt fracture test jigs fabricated according to the mechanism applied to FEA. First, the vertical shear jig was fabricated to induce the shear fracture of the bolt under a vertical force, as shown in Figure 14. The 45° combined jig was fabricated to induce the fracture of the bolt by generating tensile and plane strain stress states, while the fastened joint is widened, as shown in Figure 15. For both jigs, the insert diameter was set to 11 mm so that the M10 bolt could be mounted.

In this study, the bolt fracture test was conducted by mounting the shear jig and 45° combined jig on the universal testing machine (UTM) used in Section 2.2 to acquire load–displacement curves. In this instance, the load was obtained from the load cell in the UTM, while the displacement was acquired using DIC. If the displacement is extracted from the UTM, the stroke of the operating jig can be extracted. However, the displacement of the stroke obtained from the UTM cannot be compared because the FE modeling simplified and modeled only the inserts where the bolt is fastened and does not consider all the components installed within the UTM, as described in Section 4.1. In addition, the stroke extracted from the UTM implies that the amount of movement in the grip section is different from the insert displacement due to the absence of a reference point for the displacement calculation. Therefore, it is necessary to use the DIC technique to measure the actual displacement of the insert.

**Figure 16:**
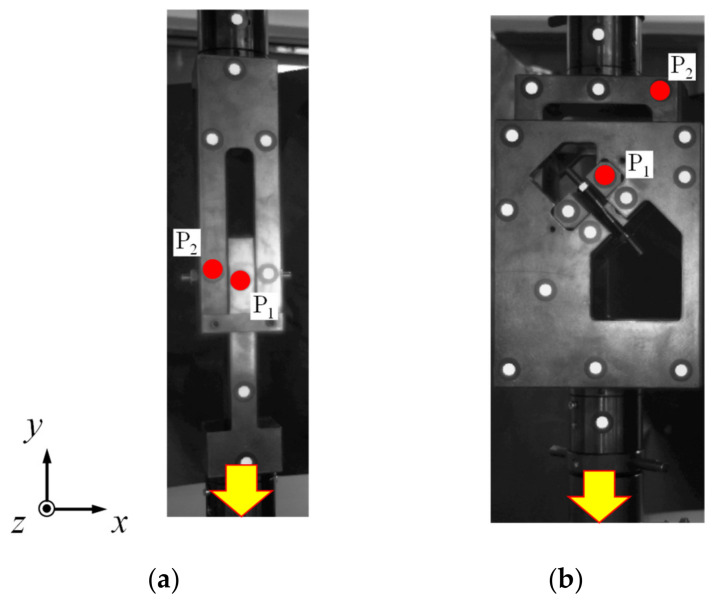
Measurement of the insert displacement using DIC (**a**) 90° shear jig (**b**) 45° combined jig.

Two reference points (P_1_ and P_2_) were attached to specific insert positions to identify the reference points and calculate the difference in the distance of movement between the points using DIC, as shown in Figure 16. The positions, which were the same as the points designated for extracting the stroke in FEA, were proposed to secure similar values.

### 4.3. Experiment vs. FEA

**Figure 17:**
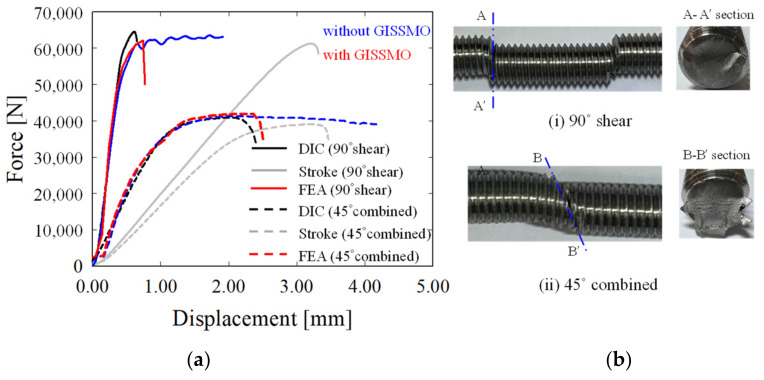
(**a**) Comparison of load–displacement curves according to the displacement extraction method. (**b**) Bolt fracture section being tested.

Figure 17a shows the load–displacement curves derived from calculating the difference in displacement in the y-direction between P_1_ and P_2_ measured through DIC. They were compared with the curves calculated from the UTM stroke and the FEA results. Figure 17b shows the bolt fracture section being tested. The solid lines represent the results extracted through the vertical shear jig, while the dotted lines show the results confirmed through the 45° combined jig test. The black curve calculated through DIC showed a higher slope than the gray curve measured through the UTM stroke. This indicates that the slope is different depending on the displacement extraction method and that the UTM stroke measurement method overestimates the actual displacement. In addition, the load–displacement curves without the application of GISSMO in FEA are marked in blue, and the curves with the application of GISSMO are marked in red. When they were compared with the DIC measurements, it was found that the results obtained by applying GISSMO were similar to the experimental curves. Finally, it was confirmed that bolt fracture could be predicted by applying the GISSMO parameters obtained above.

In this study, the fracture properties of the fastening bolt for radioactive waste containers were acquired through the application of GISSMO, and it was verified that accurate data could be obtained when applying the DIC method for which the fracture test displacement data are extracted. By identifying bolt fracture properties, the fracture of the bolt and waste container can be predicted through simulation, further enabling more accurate analysis and design in consideration of the safety of containers.

## 5. Conclusions

The fracture properties of bolts used in radioactive waste transport containers were acquired using the generalized incremental stress state-dependent damage model (GISSMO) a phenomenological damage model. In addition, jigs that can simulate the stress state in the event of the fracture of the bolt were fabricated, and the experiment and analysis results were compared for verification. The conclusions of this study are as follows.

(1) Three specimens that represent the uniaxial tensile, shear, and plane strain stress states were designed to acquire the properties of the bolt used in radioactive waste transport containers. The stress states of the specimens were calculated using LS-DYNA, and it was confirmed that an appropriate stress state was reached for all the specimens.
(2) GISSMO parameters were acquired for the UT, SH45 and N5 specimens using LS-OPT, and they were verified by applying the parameters to the bolt. Since the radioactive waste transport container is large and heavy, jigs that can simulate the fall situation of the container were designed. After examining the stress state in the bolt fracture test, the accuracy of fracture prediction was verified.
(3) The load–displacement curves of finite element analysis (FEA) were compared with those acquired in the experiment for verification. When the displacement was extracted from the bolt fracture test, the displacement calculated through the stroke of the universal testing machine (UTM) was overestimated when compared to FEA, thereby showing a small gradient. Since various interferences of the UTM cannot be considered in FEA, it is necessary to accurately capture the displacement of the insert using the digital image correlation (DIC) measurement method for an accurate comparison.
(4) The load–displacement curve of FEA with the application of GISSMO, until the point of bolt fracture, was compared to the curve obtained from the experiment, from which a good agreement was observed. In addition, it is expected that research that considers both strain rate and screw thread can be conducted using GISSMO, a fracture model used in this paper, and the DIC method introduced for actual displacement measurement. In the previous stage of these applied studies, this thesis is judged to be of academic value.

## Figures and Tables

**Table 1:** Material properties of SNB7.

Property	Value
Yield strength [MPa]	823.01
Ultimate tensile strength [MPa]	1036.38
Elastic modulus [GPa]	210.00
Poisson’s ratio	0.29
Density [g/cm^3^]	7.80

**Table 2:** Triaxiality (*η*) for different stress states.

Stress State	Triaxiality (*η*)
Shear (SH45)	0.000
Uniaxial Tension (UT)	0.333
Plane strain (N5)	0.567

**Table 3:** Specification of the stereo camera.

Digital Image Correlation Camera	
Full resolution	1936 × 1216 pixel
Frame rate at full resolution	maximum 100 fps
Minimum exposure	0.1 ms
ISO (12232 SAT method)	mono
Pixel bit-depth	12 bit
Camera control	ARAMIS professional 2020

**Table 4:** Specification of universal testing machine (UTM).

ZWICK ROELL Z100		
Load cell	capacity	100 kN
	resolution	from 0.2 kN class 1
	accuracy	±0.1% of full scale
Testing speed	0.0005~600 mm/min	
Crosshead measurement resolution	0.0083 µm/impuls	
Crosshead speed accuracy	±2 µm	

**Table 5:** Damage parameters calculated from LS-OPT.

Parameters	*m*	*f*	*ε_u_*_,SH45_	*ε_f,_*_SH45_	*ε_u_*_,UT_	*ε_f_*_,UT_	*ε_u_*_,N5_	*ε_f_*_,N5_
value	5.930	4.110	0.050	0.080	0.024	0.500	0.010	0.020

## Data Availability

Not applicable.

## References

[1] IAEA (2018). Regulations for the Safe Transport of Radioactive Material.

[2] IAEA (2014). Advisory Material for the IAEA Regulations for the Safe Transport of Radioactive Material.

[3] IAEA (2006). Management of Problematic Waste and Material Generated during the Decommissioning of Nuclear Facilities.

[4] Marsh A.I., Williams L.G., Lawrence J.A. (2021). The important role and performance of engineered barriers in a UK geological disposal facility for higher activity radioactive waste. Prog. Nucl. Energ..

[5] Pérot B., Jallu F., Passard C., Gueton O., Allinei P.G., Loubet L., Estre N., Simon E., Carasco C., Roure C. (2018). The characterization of radioactive waste: A critical review of techniques implemented or under development at CEA, France. EPJ Nuclear Sci. Technol..

[6] Cai W.Y., Jiang J., Li G.Q., Wang Y.B. (2021). Fracture behavior of high-strength bolted steel connections at elevated temperatures. Eng. Struct..

[7] Liao R., Sun Y., Liu J., Zhang W. (2011). Applicability of damage models for failure analysis of threaded bolts. Eng. Fract. Mech..

[8] Cockcroft M.G., Latham D.J. (1968). Ductility and the workability of metals. J. Inst. Met..

[9] Brozzo P., Deluka B., Rendina R. A new method for the prediction of formability limits in metal sheets. Sheet metal forming and formability. Proceedings of the 7th Biennial Conference of the International Deep Drawing Research Group.

[10] Oyane M., Sato T., Okimoto K., Shima S. (1980). Criteria for ductile fracture and their applications. J. Mech. Work Technol..

[11] Johnson G.R., Cook W.H. (1985). Fracture characteristics of three metals subjected to various strains, strain rates, temperatures and pressures. Eng. Fract. Mech..

[12] Gurson A.L. (1977). Continuum theory of ductile rupture by void nucleation and growth. J. Eng. Mater. Technol..

[13] Kuczynska M., Schafet N., Becker U., Métais B., Kabakchiev A., Buhl P., Weihe S. (2017). The role of stress state and stress triaxiality in lifetime prediction of solder joints in different packages utilized in automotive electronics. Microelectron. Reliab..

[14] Kim Y., Zhang S., Grolleau V., Roth C.C., Mohr D., Yoon J.W. (2021). Robust characterization of anisotropic shear fracture strains with constant triaxiality using shape optimization of torsional twin bridge specimen. CIRP Ann.-Manuf. Technol..

[15] Kõrgesaar M. (2019). The effect of low stress triaxialities and deformation paths on ductile fracture simulations of large shell structures. Mar. Struct..

[16] ARAMIS (2018). Manual Aramis Professional 2018.

[17] Hollomon J.H. (1945). Tensile deformation. Aime. Trans..

[18] Neukamm F., Feucht M., Haufe A. (2008). Consistent damage modelling in the process chain of forming to crashworthiness simulations. LS DYNA Anwend..

[19] Neukamm F., Feucht M., Haufe A. Considering damage history in crashworthiness simulations. Proceedings of the 7th European LS-DYNA Conference.

[20] Effelsberg J., Haufe A., Feucht M., Neukamm F., Du Bois P. On parameter identification for the GISSMO damage model. Proceedings of the 12th International LS-DYNA Users Conference.

[21] Craig K., Goel T., Eggleston T., Basudhar A., Roux W., Stander N. (2015). LS-OPT User’s Manual.

